# Overweight and Obesity are Potential Risk Factors for Disrupted Nocturnal Sleep in Iranian Adults: A Cross-Sectional Study

**DOI:** 10.3389/ijph.2021.633183

**Published:** 2021-10-29

**Authors:** Susan Darroudi, Payam Sharifan, Parastoo Sadeghzadeh, Negin Namjou, Mohammad Zamiri Bidary, Parvin Zamani, Habibollah Esmaily, Gordon A. Ferns, Mohsen Moohebati, Majid Ghayour-Mobarhan

**Affiliations:** ^1^ Student Research Committee, International UNESCO Center for Health-Related Basic Sciences and Human Nutrition, Mashhad University of Medical Sciences, Mashhad, Iran; ^2^ Department of Nutrition, Faculty of Medicine, Mashhad University of Medical Sciences, Mashhad, Iran; ^3^ Student Research Committee, Faculty of Medicine, Mashhad University of Medical Sciences, Mashhad, Iran; ^4^ Department of Medical Biotechnology, Faculty of Medicine, Mashhad University of Medical Sciences, Mashhad, Iran; ^5^ Social Determinants of Health Research Center, Mashhad University of Medical Sciences, Mashhad, Iran; ^6^ Brighton and Sussex Medical School, Division of Medical Education, Brighton, United Kingdom; ^7^ Cardiovascular Research Center, School of Medicine, Mashhad University of Medical Sciences, Mashhad, Iran; ^8^ Metabolic Syndrome Research Center, Mashhad University of Medical Sciences, Mashhad, Iran

**Keywords:** obesity, nightly sleep, nocturnal sleep, nightly sleep deprivation, MASHAD study

## Abstract

**Objectives:** Obesity is a risk factor for several chronic conditions, including sleep disorders. We aimed to analyze the relationship between BMI, body fat percentage (FAT%), hip and waist circumference, and weight on the duration of nocturnal sleep.

**Methods:** This study was part of the MASHAD cohort study. In all participants BMI and FAT% were measured. BMI was used to categorize individuals as obese, overweight, and normal subjects. FAT% was used to categorize individuals into tertile: tertile 1 (low) < 27.5, tertile 2 (medium) 27.5–41, and tertile 3 (high) > 41. The level of nightly sleep duration was categorized into three groups: <6, 6–8 (reference group), and >8 h.

**Results:** There was a significant inverse association between body weight and duration of sleep (*p* < 0.05). Obese and overweight participants had 1.152 OR (CI:1.083–1.225) and 1.126 OR (CI:1.063–1.194) for a short duration of nocturnal sleep, respectively, relative to those with a normal BMI.

**Conclusion:** BMI was an independent determinant of nocturnal sleep duration; obesity and overweight may have negative consequences on sleep duration. Weight control should be considered as a factor in adjusting sleep quality.

## Introduction

Sleep deprivation is an important public health concern [1]. The third edition of the International Classification of Sleep Disorders (ICSD-3) defines insufficient sleep as a curtailed sleep pattern on most days of the week that has persisted for at least 3 months, along with complaints of sleepiness throughout the day [2]. Unfortunately, sleep duration on workdays has declined by approximately 3.7 min per year in the last decade [3]. In a survey in 2013, an estimated 83.6 million adults in the United States were reportedly sleeping less than 7 h a day [4]. Reduced sleep duration has been found to be linked to 7 of the 15 leading causes of death in the U.S. including cardiovascular diseases, malignant neoplasms, cerebrovascular diseases, accidents, type 2 diabetes mellitus (T2DM), septicemia, and hypertension [1, 5]. Also, short sleep duration was reported to be linked to obesity. Obesity increases the likelihood of various diseases and conditions, particularly cardiovascular disease, type 2 diabetes, obstructive sleep apnea, certain types of cancer, osteoarthritis, and depression [6].

The connection between sleep duration and obesity has been established to be bidirectional in different studies. There is evidence that the relationship between BMI and sleep duration is stronger in children than adults. By aging, their association is weakened [7], and this is reported as a U-shaped relationship [8]. Most of the studies have focused on the effect of short sleep duration on obesity [9–13]. A cohort study reported that shortening sleep causes obesogenic behaviors, lower physical activity, and desire for a carbohydrate-rich diet [14], and sleep deprivation may cause neurohormonal changes leading to increased caloric intake [8]. Not much research has been done on the effect of obesity on sleep duration. In a cross-sectional study on adolescents of 11–16 years of age, it was found that those with a higher BMI had a shorter sleep duration. But the data was collected from a small sample in a specific age group, and sleep disturbance was not directly related to obesity [15]. In another study that observed women for 32 years in Gothenburg, there was a relationship between obesity and sleep problems, but the difference between genders was not considered [16], and the independent effect of obesity on sleep is still unclear [8].

Examining the association between obesity and sleep duration, most of the studies have focused on the effect of short sleep on body fat percentage. But the relationship of adiposity on sleep duration has not been assessed, especially without age and gender restrictions. Thus, we aimed to investigate the association of BMI and body fat percentage on sleep duration in a sample from the population of Mashhad, Iran.

## Methods

This study used subjects recruited as part of the MASHAD (Mashhad stroke and heart atherosclerotic disorder (MASHAD) study) cohort study; recruitment began in 2010 and finished in 2020. The details of the MASHAD cohort study have been described previously [17]. The MASHAD study sample was identified using a cluster-randomized methodology. The study was approved by the Ethics Committee of the Mashhad University of Medical Sciences (MUMS) (MASHAD study code: 85134) [17]. Participants provided written informed consent and their height (in cm), weight (in kg), waist circumference, and hip circumference were measured. Height, waist, and hip circumference were measured in centimeters to the nearest 0.1 cm with a stadiometer (SECA 217, Hamburg, Germany), and weight was measured using a calibrated digital balance in kilogram scale (SECA 813, Hamburg, Germany) to the nearest 0.1 kg. BMI was calculated using the weight [kg)/(height (m)]^2^ formula. FAT% (PBF) was assessed using a bipolar bioimpedance analyzer, TANITA BC 418model (BIA). All participants had fasted for 14 h and avoided vigorous activities during the last 12 h. BMI was categorized to obese subjects (BMI ≥30 kg/m^2^), overweight subjects (BMI: 25–29.99 kg/m^2^), and normal participants (BMI <25 kg/m^2^). FAT% was categorized as tertile, tertile 1 (low) < 27.5, tertile 2 (medium) 27.5–41, and tertile 3 (high) > 41. The level of nightly sleep duration was assessed by a questionnaire (self-reported) [17] and answers were used to categorize participants into three groups: < 6, 6–8 (reference group), and >8 h. This is in line with the Nishiura et al. study [18]. Individuals who were night workers were determined by a self-questionnaire and excluded from the study.

Statistical analysis was undertaken by SPSS version 18 (SPSS Inc. Chicago, IL, United States). The normality of the data was checked using the Kolmogorov-Smirnov test. Data presented as mean ± SD and frequency. Analysis of variance (ANOVA) and chi-square was undertaken. Multinomial logistic regression was used to evaluate the odds ratio (OR) of nightly sleep with BMI and FAT% categorized. All the analyses were two-sided and a *p*-value < 0.05 was considered significant. The figures were depicted by GraphPad Prism 6.

## Results

**FIGURE 1:**
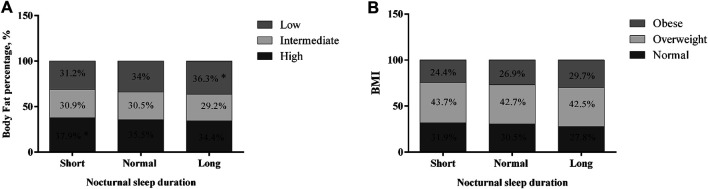
Prevalence of body fat percentage and obesity according to nocturnal sleep duration; *p < 0.05; Mashhad stroke and heart atherosclerotic disorder (MASHAD) study, Iran, 2007–2017.

Anthropometric features of the study population are summarized in Table 1. Of the 9,360 participants in this study, 36.6% had short nightly sleep duration (<6 h), 53.4% had normal nightly sleep (6–8 h), and 10% had long nightly sleep (>8 h). The mean ages of the three groups were 49.29 ± 7.97, 47.33 ± 7.99, and 46.65 ± 8.13 years, respectively. The age, weight, BMI, and FAT% were higher in subjects with short nightly sleep duration compared to the other groups (*p* < 0.05). The prevalence of short nightly sleep among study subjects was 38 and 35.7% in males and females, respectively, whereas the prevalence of long nightly sleep was 8% in males and 11.3% in females. There were significant differences between men and women in short and long nightly sleep (*p* < 0.05). The prevalence of short nightly sleep in men was higher than in women, and the prevalence of long nightly sleep in women was more than in men. Prevalence of high FAT% and overweight and obesity (based on BMI) according to nightly sleep are shown in Figure 1. There were significant differences between short nightly sleep with high FAT% (37.9%) and long nightly sleep with low FAT% (36.3%) (*p* < 0.015) (Figure 1A).

**TABLE 1:** Anthropometric features of the study subjects according to nocturnal sleep duration; Mashhad stroke and heart atherosclerotic disorder (MASHAD) study, Iran, 2007–2017.

		Short (<6)	Normal (6–8)	Long (>8)	*p*-value
Frequency, % (9,360)		3,424 (36.6%)	4,999 (53.4%)	937 (10%)	
Age, years		49.29 ± 7.97	47.33 ± 7.99	46.65 ± 8.13	<0.001
Sex	Male	1,429 (38%)^*^	2027 (54%)	300 (8%)	<0.001
	Female	2000 (35.7%)	2,972 (53%)	637 (11.3%)^*^	
Weight, kg		72.14 ± 12.93	71.9 ± 12.87	70.17 ± 12.39	<0.001
Waist circumference, cm		94.88 ± 11.97	95.33 ± 11.99	95.85 ± 12.27	0.055
Hip circumference, cm		103.5 ± 9.54	103.88 ± 9.23	103.9 ± 9.29	0.15
BMI, kg/m^2^		28.12 ± 4.75	27.82 ± 4.72	27.57 ± 4.74	0.001
FAT%		34.23 ± 10.29	22.96 ± 10.39	34.89 ± 10.32	0.039

Data are presented as Mean ± Standard Division or percentage. One-way ANOVA (analyses variance) has been done.

In obese subjects, the prevalence of short, normal, and long nightly sleep was 29.7, 26.9, and 24.4%, respectively (Figure 1B). There were significant differences between short nightly sleep in obese subjects (24.4%) and long nightly sleep in normal-weight subjects (27.8%) (*p* < 0.05).

**FIGURE 2:**
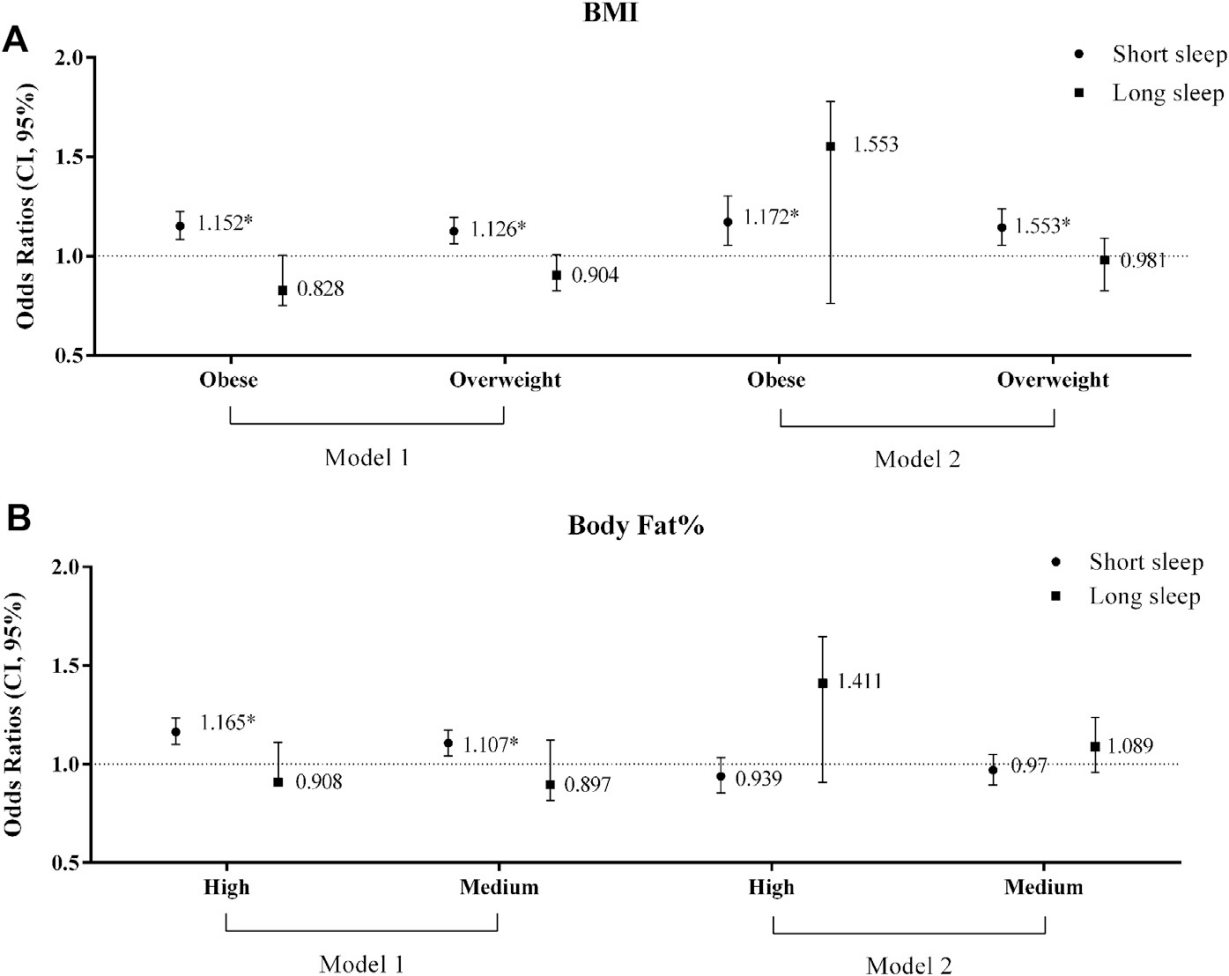
Risk of insomnia according to BMI (body mass index) **(A)** and tertile of body fat percentage **(B)** (medium: 27.5–41, high> 41); Model 1: unadjusted OR; Model 2: adjusted OR (Odds Ratio) by age and sex. Unadjusted and adjusted odds ratios (95% CI) were calculated using multinomial logistic regression. *p < 0.05; Mashhad stroke and heart atherosclerotic disorder (MASHAD) study, Iran, 2007–2017.

Multinomial logistic regression was performed to find the Odds Ratio (OR) of association between nightly sleep duration and BMI and FAT% (Figure 2). As shown in Figure 2, in comparison to normal-weight subjects, obese and overweight participants had 1.152 OR (CI: 1.083–1.225) and 1.126 OR (CI: 1.063–1.194) for short nightly sleep. In addition, when the crude results were adjusted for age and sex, this increased by approximately 1.9 percent to 14.4 and 17.2 percent for overweight and obese subjects, respectively. Based on results, third and second tertiles of FAT% had 1.165 OR (CI: 1.101–1.234) and 1.107 OR (CI: 1.042–1.175), respectively; the results were no longer significant after adjustment for age and sex (*p* > 0.05) (Figure 2). There were no significant differences between long nightly sleep and BMI and FAT% (*p* > 0.05).

## Discussion

A reduction in sleep duration is associated with an increased risk of psychological or physical problems, like depression or some chronic conditions such as coronary artery disease, hypertension, arrhythmias, diabetes, and metabolic disorders [19, 20].

Our results indicate that overweight and obese people are 12.6 and 15.2 percent more likely to have short sleep duration, respectively, compared with people with normal weight. In addition, when the raw data were adjusted for age and sex, this increased by approximately 1.9 percent to 14.4 and 17.2 percent for overweight and obese subjects, respectively. These results might be influenced by obstructive sleep apnea, a condition leading to diminished sleep quality and sleep duration, of which obesity is a substantial risk factor [21]. Furthermore, reduced physical activity or hormonal changes in obese/overweight people could be considered as causes for reduced duration and quality of sleep. The findings of a study in Gothenburg, which observed women for 32 years, showed a significant negative correlation between BMI and hours slept per night (CC −0.06, *p* = 0.03) [16].

Our findings suggest that FAT% is not an independent risk factor for short nocturnal sleep. In the initial analysis with an unadjusted model, participants with high and medium FAT% experienced short nightly sleep considerably more (16.5 and 10.7% for high and medium FAT% groups, respectively) compared with the reference population (Normal weight group). After adjusting for sex and age, the effect of FAT% on nightly sleep was not significant. The impact of FAT% on long nightly sleep was not significant in either unadjusted or adjusted results. Our results showed that FAT% could be an influential factor in short nocturnal sleep, although it has not shown to be a significant independent effect. This difference in results could be explained by gender differences in sleep, sex differences in circadian rhythmicity, or diminished association of sleep and obesity with age. In a cohort study, St-Onge et al. found no significant association between these two variables in either men nor women [22]. In a sample from Quebec, men and women who reported sleeping 7–8 h/night had a lower body fat percentage compared to those reporting 5–6 h of sleep per night [13]. In another clinical trial, total sleep time was positively associated with body fat mass [23]. These differences in similar studies can be explained by the small sample size or driving the sample, which limits the generalizability of the results to the whole population.

To our knowledge, the effect of FAT% on nightly sleep duration has not been reported. In a cohort study on adult Swedish women over 10 years, long habitual sleep was correlated with an increased incidence of obesity (BMI ≥30 kg/m^2^) compared to habitual sleep duration [24]. Long-duration sleepers showed more sedentary behaviors, less physical activity, and less dietary fiber intake in addition to a greater proportion of snacks with meals in comparison with normal sleepers [25].

In our study, the impact of waist and hip circumferences on nocturnal sleep duration was not significant. In a cross-sectional study, which was done on adolescents aged 12–13 years in China, insufficient sleepers had higher hip circumference, and the age range in this study was specific [26]. In similar studies, hip circumference was not considered a criterion for classifying participants into obese and non-obese groups, and instead, they had used waist/hip ratio [16, 23]. We found hip circumference as an independent factor for being obese. Other anthropometry measurements can be used to identify obesity, such as arm and thigh circumferences, in studying the relationship between obesity and sleep duration [27, 28]. A cross-sectional study in 2017 showed that sleep duration was negatively associated with BMI and waist circumference, in which participants had 0.9 cm lower waist circumferences per additional hour of sleep [29]. Our study was undertaken on a large sample of adults. Another study in 2016 analyzed the relationship after adjustment for age and sex and found that participants with short sleep (≤7 h/night) had significantly higher BMI, FAT%, and waist circumference when compared with other groups (*p* < 0.05), but the participants in this research were only 559 youths aged 14–28 years [30].

Interestingly, our study found that a short sleep duration (less than 6 h) is more prevalent in men (38.04%) compared to women (35.65%), and women (11.35%) experience long sleep duration (more than 8 h) more than men (7.98%). Our findings are consistent with a sample of normal sleepers of a wide age range, but this study included non-obese adults only [31]. These results might be related to sex differences in circadian timing and the influence of sex steroids on sleep [32]. Along with physiological differences, cultural differences may also influence sleep duration. Men may encounter more economic pressures and work longer hours, so this is one reason for their shorter nocturnal sleep duration than women.

Another finding was that older adults have shorter sleep duration than others. A study in 2015 which observed age and gender variations of sleep in subjects without sleep disorders, who underwent polysomnographic (PSG), showed that women slept on average 26 min longer than men and overall sleep duration diminished by only 28 min across age groups (40–50, 50–60, 60–70, and 70–80 years old.) [33]. So, following prior studies and due to the large sample size of ours, we found that sleep duration and aging are negatively associated, but we cannot explain the mechanism. A review in 2017 about sleep and human aging stated that there was some evidence that sleep need is reduced in older adults, including that older adults innately get less sleep, report less subjective sleepiness under sleep restriction conditions, and show less intense rebound sleep follow deprivation [34].

The main limitation is that, although they demonstrate the correlation between BMI and length of sleep, this study cannot prove its causality; however, a relationship between the two analyzed elements can be hypothesized.

In conclusion, BMI but not body fat percentage is an independent determinant of nocturnal sleep duration. Although the large sample size of this study is one of its strengths, prospective and intervention studies will still be necessary to prove causality and the impact of sleep duration and quality on the adult population’s physical and mental health and their work efficiency.

## Data Availability

The datasets presented in this article are not readily available because data regarding this study belongs to our institution. They do not allow researchers to share its data. Requests to access the datasets should be directed to ghayourm@mums.ac.ir.
